# Embitterment: The Nature of the Construct and Critical Issues in the Light of COVID-19

**DOI:** 10.3390/healthcare8030304

**Published:** 2020-08-27

**Authors:** Avinash De Sousa, Russell D’souza

**Affiliations:** International Institute of Organisational Psychological Medicine, Melbourne 3207, Australia; russell.f.dsouza@gmail.com

**Keywords:** embitterment, posttraumatic embitterment disorder, psychotherapy, COVID-19, pandemic, workplace

## Abstract

Embitterment is a construct that is vast and complex and may be seen in a variety of conditions and circumstances. The following paper reviews the construct of embitterment and tries to explain the concept from a psychological perspective. It also looks at the role of embitterment in the genesis of psychiatric disorders like depression, anxiety, and PTSD, while focusing on the nature and factors needed to make a diagnosis of Posttraumatic Embitterment Disorder. Situations due to the current pandemic where embitterment may develop are discussed and this is coupled with a need to manage embitterment when it develops, as it may progress to a chronic condition where its effects may be both physical and psychological. Embitterment and its management from a psychotherapy standpoint is also discussed and the same is done from a workplace perspective.

## 1. Introduction

The COVID-19 pandemic has caused havoc all over the world since January 2020 and this has led to multiple mental health effects worldwide. In fact, it has been hypothesized that there would be a second pandemic of mental health issues that would ensue after the current COVID pandemic [1]. There has been a huge surge in cases of depression, anxiety, trauma related to COVID, domestic violence, marital issues, and multiple other psychological problems worldwide. Embitterment (EMB) is by no means a new concept. It is a construct that is over a decade old and has been a part of the literature that surrounds trauma, posttraumatic stress disorder, workplace mental health, and chronic stress and adjustment. It is a specific form of adjustment disorder that may be seen in light of severe trauma, anger, feelings of resentment, and during recovery from trauma [2]. 

EMB is also an important facet of organizational psychology and workplace mental health when one believes that there is a chance that they may be cheated at the workplace, there is a chance of impending dismissal, and there is also a chance that their promotion may be halted and rather, they may be devalued and demoted to a lower position. This comes up from the feeling of being devalued at the workplace. It can also stem when one is cheated and devalued in one’s own family amongst friends and in close personal relationships [3]. The loss of productivity and self-esteem cause by EMB is immense and many cases experiencing EMB are bound to rise in light of the current COVID-19 pandemic and the fact that jobs are being lost, workplaces are being closed, and close family members are getting into conflict. The current paper explores the concept of EMB, the nature of the construct, and explains why it is important in light of the COVID-19 pandemic [4].

## 2. History of the Concept and Its Evolution

The concept of EMB came to the fore when the German research team led by Linden evaluated people from Germany a decade after the fall of the Berlin wall. They interviewed and psychologically assessed people and found that there was huge degree of resentment, anger, loss of prestige, sense of non-belonging, loss of economy, and societal stigma faced as a result of the German unification. They all experienced serious prolonged feelings of psychological distress and the event of the unification and subsequent events caused them to feel frustration, humiliation, and other derogatory feelings. This was an important facet as very often EMB stems from a major trigger event, in this case being the unification of Germany. There is also a need to understand that similar emotions have been experienced in cases of economic recessions, natural tragedies, and certain calamities [5]. 

The concept of EMB has been defined by Linden as “a distinct state of mood known to everyone. It can be seen in the context of exceptional though “normal” negative life events. It is an emotional reaction e.g., to humiliation, to being severely disappointed by others, or to violations of basic values” [6].

EMB has evolved since then and has been expanded to encompass posttraumatic symptoms and anxiety and depressive features, as well as potentially being a construct to which someone has genetic predisposition and may even be a personality trait. The concept of EMB has even been reflected in the Bible and certain ancient texts. The construct has, however, not been reflected a lot in psychopathology and psychiatric textbooks. The feeling of constant unfairness and bitterness has been constantly seen in therapy settings and psychotherapy explorations as well [7].

Currently, during the current pandemic and lockdown, many people who started new businesses and ventures have faced tremendous losses and financial strains and many people have lost a loved one to COVID, while there have been job losses, demotions, overwork, excess work from home, and frustration that have led to feelings of EMB creeping into many normal people, with the resultant feelings of depression and resentment imbuing [8].

## 3. Expanding the Concept of EMB

There are many situations when an individual may feel that there has been injustice coupled with emotional reactions like anger, hostility, guilt, and shame. Many people experience bitterness in life, love, and relationships, both personal and at work, and have a persistent feeling of exasperation, exhaustion, and feel that they have been targeted and blamed and are a victim, with feelings of humiliation, helplessness, hopelessness, worthlessness, and desire for revenge. EMB is caused by an event that is a trigger and by a series of events that make the individual perceive and believe that only he or she has been targeted unjustly and has had to bear the brunt of events that were beyond one’s control. The state of EMB is an emotional state on its own and is separate from depression, anxiety, stress, and disgust. It may also be a part of these conditions and may be part of the symptomatology that is experienced in these conditions [9].

It is a condition that is continuous, progressive, and does not abate easily in therapy or with medical intervention. It is also distinct from demoralization and other posttraumatic states, while it may, at times, be a part of an emotional contagion that is being experienced by the entire family or even, an entire cohort or subset of the population. 

In relationships, EMB is common after a breakup, mutual separation, or a divorce. A person may be angry or upset with the other person and feel a sense of betrayal and bitterness. There is also a strong feeling of being cheated, guilt, anguish, and hatred that ensues. One feels that one has wasted a part of one’s life and feels a grave injustice has been done. There are prolonged feelings of defeat, injustice, inability to have detected that this would happen, foolishness, and the need for getting even with the person concerned. This mental state is persistent, long lasting, and one gets flashbacks of the event, series of events, and past states that have led to this feeling [10].

Some people have the tendency to feel more embittered than others. This is due to a genetic tendency towards emotional sensitivity, extreme reactivity to being hurt, extreme responses to criticisms, and tendency to feel being a victim all one’s life. The intrusive though aspect of EMB is very similar to that experienced by patients of posttraumatic stress disorder (PTSD). The state of EMB is painful and unpleasant with a feeling of getting even; one may even feel rewarded if there is a way that revenge can be carried out for the wrong suffered [11]. 

EMB has a cognitive component as well, where the recurrent thoughts turn into ruminations and create a negative cycle of thinking where the person is mulling over the events that triggered the EMB with constant self-blame and self-anger. Many times the subject may feel that others are not as serious about this event as they are. There may be mood swings along with the EMB and they move from despair to happiness when they feel their loss can be avenged. Thus, the spectrum of psychopathology in which EMB manifests itself is so vast and complex that it can be a symptom, a part of multiple psychiatric disorders, or can be a disorder itself [12].

Studies have yielded positive associations between EMB and a personality trait called affective inhibition. Affective inhibition is a personality where there is an inability to express one’s emotions well and there is a tendency to express the same through physical symptoms, which is a concept developed in psychosomatic medicine akin to alexithymia and implicated in the pathogenesis of many medical illnesses that have been labelled psychosomatic. EMB can result in medical illness that is exacerbated and augmented by psychological undertones. This is also seen in situations from where there is no escape like a failed marriage, negative organisations, and bad jobs [13].

## 4. Posttraumatic Embitterment Disorder (PTED)

On the lines of PTSD, the construct has resulted in the development of a new concept called PTED where the core triggering emotion is EMB, whereas the core emotion in PTSD is anger. PTED is a pathological reaction to drastic life events and the trigger is an extraordinary though common negative life event like divorce, dismissal, personal insult, or vilification. The consequence is severe and long-lasting EMB. The event is too important to be ignored and assimilation of the event into existing thought schemata or basic beliefs is not possible, while at the same time, a change/adaptation of these schemata (accommodation) is unthinkable and impossible, causing a disorder of adaptation. PTED arises when basic beliefs are questioned, attacked, disproved, or degraded through a life event or the behaviour of others. This theory of violation of basic beliefs also explains why events, which seem to be trivial for some people, can be of importance to others [14].

The diagnostic criteria of PTED is as below: [15]

- Core criteria:
  1. A single exceptional negative life event precipitates the onset of the illness.
  2. The individual knows about this life event and sees their present negative state as a direct and lasting consequence of it.
  3. The individual experiences the negative life event as unjust and responds with embitterment and emotional arousal when reminded of the event.
  4. No obvious mental disorder in the year before the critical event. The present state is not recurrence of a pre-existing mental disorder.
- Additional signs and symptoms:
  1. The individual sees themselves as a victim and as helpless to cope with the event or the cause at the workplace.
  2. The individual blames themselves for the event, for not having prevented it, or for not being able to cope with it.
  3. The individual reports of repeated intrusive memories of the workplace critical event. They even think that for some parts, it is important not to forget.
  4. The individual expresses thoughts that it no longer matters how they are doing and are even uncertain whether they want the wounds to heal.
  5. Additional emotions are dysphoria, aggression, and downheartedness, which can resemble melancholic depressive states with somatic syndromes.
  6. Patients show a variety of unspecific somatic complaints such as loss of appetite, sleep disturbances, and pain.
  7. Patients can report phobic symptoms in respect to the workplace or to persons at the workplace related to the event.
  8. Energy, motivation, and drive is reduced and blocked. The individual experiences themselves not as drive-inhibited but rather as drive-unwilling or negative.
  9. Emotional modulation is not impaired, and patients can show normal affect when they are distracted or can even smile when engaged in thoughts of revenge or consummatory pleasure.
- Duration: Longer than three months
- Impairment: Reduction in performance in daily activities in the workplace and might display impaired role in the workplace.

## 5. Distinguishing PTSD and PTED

PTSD and PTED are two distinct disorders and, at the same time, are conditions that are interlinked, intertwined, and comorbid. PTED applies to experiencing, witnessing, or being directly confronted with a highly traumatic (though, unlike PTSD, not necessarily life-threatening) event or events (e.g., difficult divorce, major losses of significant others, serious illness, disability, job loss, workplace stress, physical or emotional abuse, domestic violence, sour relationships etc.), leading to chronic (longer than three months) feelings of EMB, hostility, anger, resentment, irritability or rage, and the obsessive, sometimes compelling, desire for revenge and retribution. A little embitterment may be seen in all mental disorders and is not the same as PTED [16]. 

Subtle feelings of bitterness that commonly come and go with life’s inevitable existential frustrations and disappointments are not enough to warrant a diagnosis of PTED. Patients with personality disorders like schizoid, narcissistic, and obsessive-compulsive personality disorders may show shades of PTED, as the roots of these disorders also lie in repressed anger, resentment, and rage. Unlike PTSD, where anxiety is the main component with repeated thoughts of the event that caused trauma, emotional numbing, and panic attacks when these thoughts ensue, PTED may have a feeling of wanting to be totally alone and alienated from all intimate relationships, friends, and family [17]. One becomes cynical, negative, defensive, and demotivated festered by anger, rage, resentment, and narcissistic wounding. Some people may also develop a total lack of empathy and compassion for others. The tendency to displace these feelings on people who do not deserve them also comes up [18]. 

## 6. EMB in the Context of COVID-19

There have been many instances in the context of the current pandemic that EMB and PTED may develop in people. This stems from various events that one may experience due to the pandemic and after contracting COVID. Many patients who have contracted the illness have had to bear the brunt of the stigma associated with COVID and have been rejected by the building and places where they dwell. There have been many events during COVID-19 experienced by people that may haunt them for a lifetime and lead to feelings of EMB and prompt the development of PTED [19]. Some of these situations may be [20]:

1. Loss of a loved one to COVID-19: this may cause feelings of guilt, shame, and resentment as the disorder is beyond one’s control and there is a question that why did the life of a loved one have to be lost and that no one could do anything about the same.
2. Grief for loss of a loved one: There have been instances when a family has lost a member to COVID and has not been able to see the person towards the end and even probably, not attend the funeral and burial due to restrictions, which may lead to PTED.
3. The experience of COVID: When one has COVID, there may negative experiences of being in quarantine and isolation, which may have negative implications. There may also be questions as to why did COVID develop in me and why did I have to suffer.
4. Loss of a job and demotion: COVID has resulted in an economic recession and there have been many instances when people have lost their jobs and have been demoted as well as receiving half salaries, despite working from home, and have not yet been recognized by their organizations. This can cause both EMB and PTED.

There is a need for counselling in many such situations and to help the people deal with these feelings through psychotherapy. 

### Scales for the Assessment of PTED

There are, to the best of our knowledge, only two specific scales for the measurement of EMB, one of which is known as the PTED self-rating scale. The scale was originally in German and has been translated into English. It has 19 items and is a Likert-type scale that measures various facets of PTED. The scale has good reliability and validity and has been used in a number of studies on PTED in general and specific populations. It has a good construct validity as well [15,16]. There is a scale known as the Bern Embitterment Inventory, which is in German and English, but has been used far more sparingly compared to the PTED self-rating scale [21]. 

## 7. Psychotherapeutic Management of PTED

PTED and EMB are complex conditions to manage in a psychotherapy setting due to the vast nature of the psychopathology that may lie beneath the surface and the intense feelings of bitterness, coupled with the rejection of any form of aid and perceived toxic workplace environments in many instances. The people suffering from PTED and EMB may not feel that they have a problem and may rarely seek help and visit a therapist. They may come half-heartedly when brought by a family member and may not be regular and may even be noncompliant in therapy. They may have feelings that are aggressive and fatalistic where they are not open to alternative perspectives and thought patterns expressed by the therapist and they, hence, are less amenable to change and a lot of time has to ensue before they manage to deal with these emotions [22]. 

Psychotherapy has to use principles of preventive psychiatry, build resilience, and rebuild the human capital in every person fretting with EMB. The International Institute of Organisational Psychological Medicine (IIOPM) has developed specific guidelines for psychotherapy to reduce EMB and rebuild personality. We may, thus, even prevent PTED from developing and nip it in the bud when it starts. The specific form of psychotherapy that works well in people with EMB and PTED is called Wisdom Competence Therapy (WCT), which can work at an individual level and also as group interventions at an organizational and college level [23]. Preventive programs and awareness programs about EMB and PTED in the workplace can go a long way to help people become aware and seek help in this area when needed. It is also worthwhile to mention that the therapy for PTED works in the area of the cognitive symptoms and feelings, while the socioenvironmental factors causing PTED also need modification, which is beyond the scope of this paper. 

## 8. Basic Tenets of Wisdom Competence Therapy (WCT)

WCT believes that wisdom and competence are both neurobiological and psychological constructs. The therapy combines the nuances of neuroscience, principles of cognitive behavioural science and specific tasks and activities to build wisdom, enhance personal transformation, promote inner growth, and evolve one to having a greater purpose in life. It also borrows from existential thinking in some aspects [22]. Wisdom in psychology is the ability of people to manage and deal with situations of life particularly difficult and problematic with ease, while competence in the context of WCT is the trait that helps people tolerate better and overcome situations of life in all spheres that might be particularly difficult and where one may feel helpless and no escape. IIOPM has conducted numerous workshops on WCT in the common man, education sectors, teachers, doctors, and corporate settings. It is well documented that EMB and PTED leads to a decline in work productivity, reduces creativity, disallows the retention of talent, and causes greater resentment towards the organisation and life in general [24]. 

When one looks at wisdom, it is interesting to note that wisdom is further complicated by the fact that there appears to be an inverse relationship between thinking of oneself as wise and actually being wise. Wisdom in psychotherapy is cultivated by teaching and motivating clients to express emotions and express a genuine concern for people that matter to them. The concern for others is often summarized in studies on wisdom as a concern for the consequences of our actions, in the near and long term, for those close and far. Ancient Buddhist traditions regard wisdom and concern for others (compassion) as inseparable and as two wheels of a cart. They represent in Buddhism as the coming together of the head and the heart, an integration of which is the key feature of wisdom. Developing one in the client without the other can lead to trouble in psychotherapy. Clients in therapy are also taught to have reflective thinking, journaling, and reflect on their actions along with developing an insight into the issues that plague them [25]. 

Many facets of EMB emerge in therapy where it can be [26]:

1. A normal emotion limited in intensity and duration.
2. A predisposition pattern due to genetics and certain aspects of personality.
3. A symptom that can be related to other psychological disorders.
4. The result of a chain of minor events that have accumulated, rather than a single event.
5. The result of one large life-disrupting event.
6. A cognitive pattern of thinking seen in most events that the individual will experience.
7. A disorder in the form of PTED.
8. Families and cohorts that may have EMB/PTED and cause emotional contagion.

### Future Research Needs

One needs to evaluate whether the concept and entity of PTED across all cultures and also whether the concept of EMB has variations in psychopathology across cultures. There is a need for assessing the reliability and validity of the PTED self-rating scale across various countries and it needs translation into various languages, while the notion of EMB needs to be studied in diverse organizational and workplace populations as well as in children and adolescents. 

## 9. Conclusions

Many studies shown that PTED exists along with EMB in all spheres of life and affects individual wellbeing, social life, personal thinking, and workplace productivity. The chronic nature of the condition and mixed prognosis can result in increased absence from work, disinterest in human relationships, disinterest in emotions, and a general resistance to psychotherapy. The disorder and symptoms when present have a very complex clinical picture and with high financial and economic costs to the individual and the whole family. In the context of the current pandemic, both EMB and PTED shall be very common and cause guilt, anger, shame, conflict at work, redundancy, and unemployment. There is a need for interventions at the personal level and workplace, where resilience building and valuing the human capital in all strata using the underpinnings in WCT and existential therapy can offer evidence-based solutions.
